# Single-cell RNA sequencing offers novel perspectives in viral infection research

**DOI:** 10.3389/fcimb.2026.1798303

**Published:** 2026-04-01

**Authors:** Hao Zhang, Yafei Li, Hang Li, Shaomeng Liu, Dang Wang, Huanchun Chen, Qingyun Liu, Xiangru Wang

**Affiliations:** 1 National Key Laboratory of Agricultural Microbiology, College of Veterinary Medicine, Huazhong Agricultural University, Wuhan, China; 2 Key Laboratory of Preventive Veterinary Medicine in Hubei Province, The Cooperative Innovation Center for Sustainable Pig Production, Huazhong Agricultural University, Wuhan, China; 3 Engineering Research Center of Animal Biopharmaceuticals, The Ministry of Education of the People’s Republic of China (MOE), Wuhan, China; 4 Frontiers Science Center for Animal Breeding and Sustainable Production, Huazhong Agricultural University, Wuhan, China

**Keywords:** cellular heterogeneity, host-virus interaction, scRNA-seq, transcriptomics, viral infection

## Abstract

The global emergence of multiple viral zoonoses underscores the substantial threats of viral infections to human health. Given the dynamic and complex mechanisms underlying viral pathogenesis, sophisticated approaches are requisite to advance viral research. Here, we present a systematic review of single-cell RNA sequencing (scRNA-seq), a high-throughput technology enabling transcriptomic profiling at the individual cell level, focusing on its pivotal role in elucidating heterogeneous host cellular responses to viral infection and deciphering underlying pathogenic mechanisms. We summarize scRNA-seq’s developmental milestones, compare characteristics of various platforms, and outline its key applications in viral infection research: identifying infection-induced novel cell types/subpopulations, characterizing virus-specific host cell gene expression changes, defining viral target cells, elucidating antiviral immune mechanisms, and clarifying *in vivo* viral distribution and pathogenesis. By synthesizing these information, this review offers novel research and technical perspectives for dissecting the dynamic and complex virus-host interactions, aiding future advancements in viral infection research.

## 1 Introduction

Over the past two decades, nearly all newly emerging zoonotic infectious diseases that have caused significant global damage are viral infections, such as Nipah virus disease, West Nile fever, SARS, pandemic H1N1 influenza, MERS, Ebola hemorrhagic fever, Zika virus disease, COVID-19, and monkeypox (Redd et al., 2013; Viboud and Lessler, 2018; Flerlage et al., 2021). In addition, viral diseases such as acquired immunodeficiency syndrome, Rabies, and Japanese encephalitis have posed serious threats to global human health for nearly a century, yet their pathogenic mechanisms remain incompletely understood. Elucidating the pathogenic and immune mechanisms involved in viral infections is fundamental for the effective prevention and control of infectious diseases. During infection, interactions among virus-host-environment lead to dynamic changes that collectively influence disease onset, progression, and final outcome (Ratnasiri et al., 2023). Consequently, understanding the interactions between viruses and their hosts has always been critical in deciphering viral pathogenesis.

In the past decades, sequencing technologies have been widely employed to investigate virus-host interactions. By leveraging the principle of high-throughput parallel sequencing, traditional transcriptomic sequencing techniques capture dynamic changes in gene expression of both hosts and viruses during the infection process. It provides crucial technical support for viral infection research (Metzker, 2010). However, traditional transcriptome sequencing technique pools RNA from all cells together, yielding average-level changes in gene expression profiles across all assayed cells. It fails to provide detailed information about individual cell type or subtype and could not reveal the heterogeneity within cell populations of tissues (Huang et al., 2021). Moreover, traditional transcriptome sequencing techniques struggle to dissect dynamic processes of the host such as cell differentiation or stress responses at the single-cell level, and they fail to reveal intercellular communication networks (Marioni et al., 2008). Due to limitations in sequencing depth and accuracy, traditional transcriptome sequencing techniques also have certain shortcomngs in detecting low-abundance transcripts such as rare mRNAs or long non-coding RNAs (Trapnell et al., 2014).

scRNA-seq, an advanced technique derived from traditional sequencing techniques, enables RNA sequencing at the single-cell level. It has been applied to the study of various viral infections, including those caused by SARS-CoV-2 (Ren et al., 2021), herpes simplex virus (Wyler et al., 2019; Hu et al., 2022), African swine fever virus (Zheng et al., 2022), influenza virus (Sun et al., 2020; Gao et al., 2021; Wimmers et al., 2021), and dengue virus (Jiang et al., 2023). scRNA-seq has revealed detailed transcriptomic differences between infected and uninfected cells, offering more precise data for elucidating host-virus interactions. This review systematically summarizes the developmental history of scRNA-seq and its workflow. We further highlight its applications in viral infections research from different perspective, discuss current challenges in applying scRNA-seq to virology research, and provide perspectives on its future development. It aimed to provide a comprehensive understanding of the role of scRNA-seq in deciphering of viral infection mechanisms, thereby promoting in-depth exploration of virus-host interactions.

## 2 Overview of single-cell sequencing technologies

**Figure 1:**
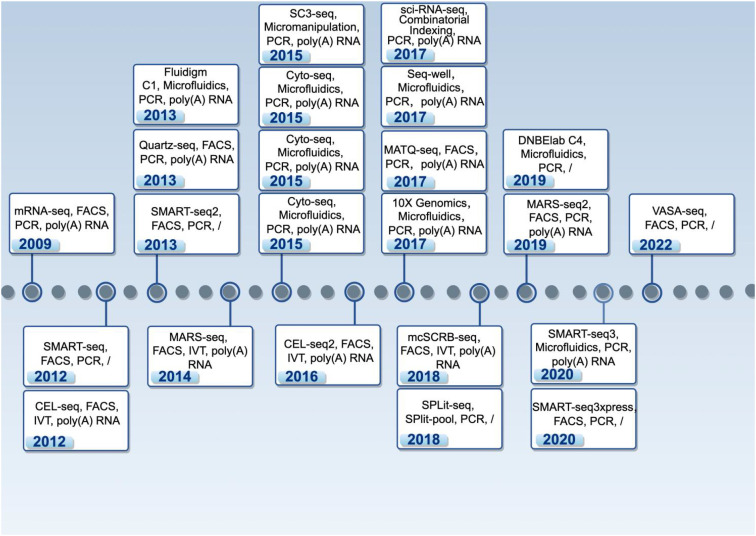
The development of scRNA-seq technology. The above information is organized according to the following categories: Platforms, Isolation strategies, Amplification methods, UMI, and Targets. This schematic illustration was generated using Figdraw, and its reproduction in this work has been obtained with formal permission. /: No specific restriction.

In 2009, Tang et al. pioneered the analysis of cDNA expression profiles in single mouse blastomeres using mRNA-seq, laying the groundwork for subsequent scRNA-seq (Tang et al., 2009; Svensson et al., 2018). In 2011, Islam et al. enabled high-throughput scRNA-seq by analyzing 92 single-cell transcriptomes via STRT-seq (Islam et al., 2011). Continuous advancements in cell isolation and bioinformatics technologies have driven the rapid emergence of novel scRNA-seq methodologies (**Table 1**, **Figure 1**). In 2012, Ramsköld et al. developed SMART-seq, a full-length mRNA-detecting scRNA-seq technique that significantly enhanced transcript coverage. However, it lacked strand specificity and exhibited inefficient transcription for sequences exceeding 4 kb (Ramsköld et al., 2012). That same year, Hashimshony et al. introduced CEL-seq, which utilized linear amplification-based sequencing to overcome insufficient RNA input in traditional single-cell methods. Although its paired-end deep sequencing enabled accurate strand detection, the technique displayed pronounced 3’-end bias (Hashimshony et al., 2012).

**Table 1:** Information of different scRNA-Seq techniques.

Platforms	UMI	Region	Advantages	Disadvantages	References
mRNA-seq	NO	3’ end	High sensitivity and good reproducibility	High cost and complex data processing	(Tang et al., 2009; Svensson et al., 2018)
SMART-seq	NO	Full-length	Enhanced sensitivity to minimize the loss of nucleic acids	Reduced throughput and bias towards certain transcript lengths	(Ramsköld et al., 2012)
CEL-seq	YES	3’ end	High sensitivity and good reproducibility	Specificity to mRNA, 3′ bias and sensitivity to small copy numbers	(Hashimshony et al., 2012)
SMART-seq2	NO	Full-length	High sensitivity and full-length transcript sequencing	High cost, technical complexity and time-consuming	(Picelli et al., 2014)
Fluidigm C1	NO	Full-length	Automated process and versatility	Low throughput and high cost	(Xin et al., 2016)
Quartz-seq	YES	3’ end	High sensitivity, reproducibility and data quality	Low throughput and high cost	(Sasagawa et al., 2013)
MARS-seq	YES	3’ end	High throughput and specificity	Low amplification efficiency	(Jaitin et al., 2014)
Cyto-seq	YES	3’ end	Cost-effective and high throughput	Intermingling of RNA molecules from different sources	(Fan et al., 2015)
Drop-seq	YES	3’ end	Cost-effective and high throughput	Limitations on cell size and type and low sensitivity	(Ziegenhain et al., 2017)
InDrop-seq	YES	3’ end	High throughput and diversity	Technical complexity	(Klein et al., 2015)
SC3-seq	YES	3’ end	Low cost and high throughput	RNA samples experiencing cross-contamination	(Nakamura et al., 2015)
CEL-seq2	YES	3’ end	High sensitivity and cost-effective	Potential for bias and sensitive to sample processing	(Hashimshony et al., 2016)
10X Genomics	YES	3’ end	High-resolution single-cell analysis and fast cycle time	Restriction of sequencing to the 3′ end only	(Zheng et al., 2017)
MATQ-seq	YES	Full-length	Efficiency and versatility	Low cell throughput	(Sheng and Zong, 2019)
Seq-well	YES	3’ end	High throughput and ease of operation	Not appropriate for analyzing variable splicing and allelic expression	(Gierahn et al., 2017)
sci-RNA-seq	YES	3’ end	Low cost, high yield and high sensitivity	Loss of cytoplasmic transcripts	(Martin et al., 2023)
mcSCRB-seq	YES	3’ end	High throughput and high sensitivity	High cost and sensitive to sample quality	(Bagnoli et al., 2018)
SPLit-seq	YES	Full-length	High throughput and no single-cell isolation required	Sensitivity limitations and potential sample contamination	(Rosenberg et al., 2018)
MARS-seq2	YES	3’ end	High sensitivity and cost-effective	Sensitivity to sample quality	(Keren-Shaul et al., 2019)
DNBElab C4	YES	Full-length	Accurate measurement	Sensitivity to sample quality	(Wang W. et al., 2021)
SMART-seq3	YES	5’ end	High sensitivity and full-length transcript sequencing	High cost and time-consuming	(Hagemann-Jensen et al., 2020)
SMART-seq3xpress	YES	Full-length	Increased cellular throughput	High cost	(Hagemann-Jensen et al., 2022)
VASA-seq	YES	Full-length	Comprehensiveness and low cost	High data complexity	(Salmen et al., 2022)

SMART-Seq, Switching mechanism at 5’ End of RNA Template; Cel-Seq, Cell Expression by Linear amplification; MARS‐seq, massively parallel single‐cell RNA‐sequencing; Cel-Seq, Cell Expression by Linear amplification; MATq-Seq, multiple annealing and dC-tailing-based quantitative single-cell RNA-Seq; IVT, *in vitro* transcription; PCR, polymerase chain reaction.

In 2013, Picelli et al. developed SMART-seq2 as an enhancement to SMART-seq. This protocol incorporated locked nucleic acids, elevated MgCl_2_ concentrations, and betaine but remained restricted to amplifying and detecting poly(A)-tailed mRNAs (Picelli et al., 2014). The Fluidigm C1 system (Xin et al., 2016), an early commercial single-cell sorting platform, employed microfluidic automation to capture ~96 cells within hours for library preparation and sequencing, substantially streamlining experimental workflows. Nevertheless, its low cellular throughput, limited sample compatibility, and high cost hindered widespread adoption.

MARS-seq was developed in 2014 (Jaitin et al., 2014), which isolated single cell based on fluorescence-activated cell sorting (FACS) and applied molecular barcoding for precise transcript counting. It features high throughput and sensitivity, allowing simultaneous detection of thousands of cells. After 2015, innovative technologies such as Cyto-seq (Fan et al., 2015), Drop-seq (Ziegenhain et al., 2017), and inDrop (Klein et al., 2015) emerged, scaling cell capture capacity to 10^4^–10^5^ cells per experiment. Cyto-seq isolates single cells by randomly distributing them into microwell arrays and pairing them with uniquely barcoded gel beads. Drop-seq and inDrop isolates single cells by co-encapsulating cells with barcoded beads in nanoliter droplets via microfluidic devices.

In 2017, Zheng et al. reported the 10 × Genomics platform (Zheng et al., 2017), which co-partitions gel beads (loaded with barcodes and primers) and individual cells within oil droplets via microfluidics. It could capture gene expression data from a large number of single cells in a single experiment. Compared to Drop-seq and inDrop, 10 × Genomics can capture a greater number of cells and genes, showing higher sensitivity and lower technical noise. Unique Molecular Identifier (UMIs) is a distinct tag to each DNA molecule composed of ~10 bp random bases.—assign, which is used for distinguishing between different samples and precise molecular quantification in pooled sequencing. UMIs have been applied in many scRNA-seq protocols, including SC3-seq (Nakamura et al., 2015), CEL-Seq2 (Hashimshony et al., 2016), MATQ-Seq (Sheng and Zong, 2019), Seq-Well (Gierahn et al., 2017), sci-RNA-seq (Martin et al., 2023), mcSCRB-seq (Bagnoli et al., 2018), and SPLiT-seq (Rosenberg et al., 2018).

## 3 The workflow of scRNA-seq

**Figure 2:**
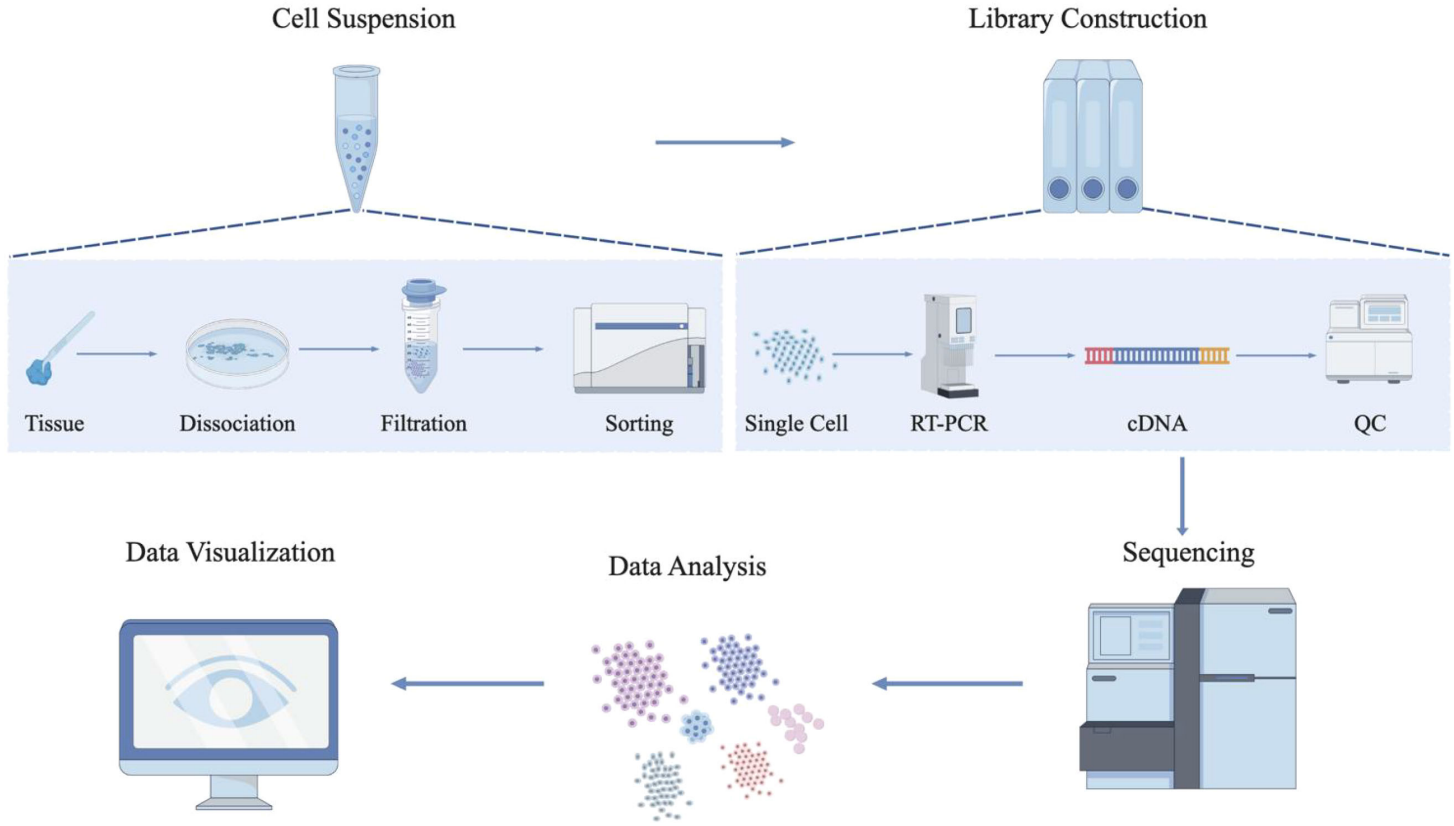
An overview of scRNA-seq protocols. The workflow of scRNA-seq typically encompasses three core experimental stages: the preparation of a high-quality single-cell suspension, the construction of sequencing libraries (tailored to scRNA-seq platforms), and high-throughput sequencing. Subsequently, computational analysis and visualization are conducted to uncover cellular heterogeneity. This schematic illustration was generated using Figdraw, and its reproduction in this work has been obtained with formal permission.

The standard workflow of scRNA-seq primarily includes single-cell isolation, reverse transcription, cDNA synthesis, single-cell library construction, high-throughput sequencing, and data analysis (de Klerk and t Hoen, 2015) (**Figure 2**). Among these, single-cell isolation is the most critical step in scRNA-seq, the tissue needs to be dissociated into a single-cell suspension (Wang et al., 2023). Traditional cell isolation methods include serial dilution, micromanipulation, FACS, and laser capture microdissection (LCM) (Liu et al., 2014; Yang et al., 2021b) (**Table 2**). Currently, cell sorting platforms based on microfluidic technology and magnetically activated cell sorting (MACS) have significantly improved the efficiency and accuracy of single-cell isolation (Zhao et al., 2016; Valihrach et al., 2018) (**Table 2**).

**Table 2:** Single cell isolation techniques.

Techniques	Throughput	Equipment	Final volume/cell	References
Microfluidic	High	Microfluidic chip, detector	Nanoliters	(Reece et al., 2016)
MACS	High	Magnetic bead, magnetic columns and separator and flow cytometer	Microliters	(Hu et al., 2016)
Micromanipulation	Low	Micropipette, microscope and micromanipulator	Microliters	(Brauns and Goos, 2005)
LCM	High	Laser system, microscope	Microliters	(Kroneis et al., 2016; Rao et al., 2022)
FACS	High	Flow cytometer	Microliters	(Gross et al., 2015; Hodne and Weltzien, 2015)

Microfluidic technology enables automated capture of individual cells using specialized integrated fluidic circuits. The captured single cells can be verified through imaging technologies to assess the capture efficiency. Subsequently, each cell undergoes a series of specific chemical reactions within a sequence of microchambers. These reactions are precisely controlled to proceed in a specific order, starting with cell lysis, followed by reverse transcription of RNA into cDNA, and concluding with PCR amplification (Reece et al., 2016).

MACS employs antibodies immobilized on the surface of magnetic beads. These antibodies can specifically recognize and bind to proteins on the target cell surface. When a heterogeneous cell suspension is subjected to an external magnetic field, labeled cells become activated by the magnetic beads and are retained, while unlabeled cells are washed away. Upon removal of the magnetic field, the retained cells can be collected through elution. This method achieves high-purity cell isolation.

The analysis of scRNA-seq data typically (Bagnoli et al., 2018) involves alignment of sequencing reads, quality control, removal of technical noise, data normalization, differential gene expression analysis, cell clustering, dimensionality reduction, and trajectory inference (Waickman et al., 2021). First, tools such as TopHat2 and STAR are used to process single-cell RNA sequencing data into the “fastq” format, which stores biological sequencing data including nucleotide sequences and their associated quality scores (Kim et al., 2013; Dobin and Gingeras, 2015; Srivastava et al., 2016; Prieto et al., 2022). Data quality control is initially performed using tools such as FastQC, SinQC, and Scater to evaluate fundamental parameters including sequencing error rates (<1%), sequence duplication (<30%), and GC content (40%-60%), thereby ensuring the reliability and accuracy of subsequent analytical procedures. For multi-batch viral infection sequencing data, the scRNABatchQC tool is further applied to enable simultaneous comparison of technical and biological characteristics across multiple datasets. This approach facilitates the identification of systematic biases, batch effects, and outliers, and allows for the effective discrimination between technical artifacts and genuine biological variation (McCarthy et al., 2017; Yang et al., 2017; Jiang, 2019; Liu et al., 2019). Intra-sample and inter-sample data normalization is conducted using tools including SCTransform, BasiCS, Scran, and Linnorm. Normalization allows data from different samples or different cells within the same sample to be compared and analyzed under a unified standard (Adil et al., 2021). Batch effect correction—essential for minimizing non-biological variability caused by differences in experimental conditions, processing times, or sequencing platforms—is performed using methods including Mutual Nearest Neighbors (MNNs), Canonical Correlation Analysis (CCA), kBET, and REBET (Butler et al., 2018; Tran et al., 2020; Yang et al., 2021a; Yu et al., 2023). scPLS can be utilized to preserve biological signals relevant to viral infection (Chen et al., 2020). The multimodal integration model of scbean allows for the simultaneous correction of batch effects and modality differences, rendering it appropriate for multi-omics data integration in viral infection research (Li et al., 2021). Harmony or the BBKNN algorithm implemented in Scanpy is recommended, as it offers a favorable balance between correction efficiency and accuracy. Additionally, the scVI deep learning model can be employed, which takes batch information as a conditional variable to learn the latent distribution of the data and enable more accurate batch integration (Butler et al., 2018).

Given that single-cell datasets typically contain expression information of tens of thousands of genes, direct analysis can be computationally intensive and complex. Dimensionality reduction techniques such as Principal Component Analysis (PCA), t-Distributed Stochastic Neighbor Embedding (t-SNE), and Uniform Manifold Approximation and Projection (UMAP) are thus applied to transform high-dimensional data into more interpretable and visualizable forms. Analyses of cellular heterogeneity and differential gene expression help uncover transcriptional regulation mechanisms and the biological relevance of cellular diversity (Becht et al., 2018; Miao et al., 2018; Vans et al., 2021). Common scRNA-seq analysis methods, software packages, and their functions are listed in **Table 3** (Penaranda and Hung, 2019).

**Table 3:** scRNA-Seq analytical workflows and software.

Method	Software package	Function	References
Read mapping	Kallisto, STAR and UMI-tools	Splicing, expression quantification, and UMI processing	(Dobin and Gingeras, 2016; Smith et al., 2017)
Quality control	SoupX, scrublet, DoubletDecon and CellBender	Focusing on removing artifacts that confound data analysis and interpretation	(DePasquale et al., 2019; Young et al., 2020)
Data integration, normalization, visualization and clustering	Seurat, Scate and Garnett	Data integration, normalization, visualization and clustering	(Hie et al., 2019; Michielsen et al., 2021)
Enrichment analysis	ClusterProfiler, singleseqgset and topGO	Functional interpretation using GSEA, KEGG, and GO	(Consortium, 2017; Kanehisa et al., 2017)
Cell type annotation	SingleR, CHETAH, SCINA	Cell type identification and unbiased clustering	(Aran et al., 2019; Zhang et al., 2019)
Trajectory analysis	Monocle, TradeSeq	Pseudotime analysis to reveal dynamic gene expression changes during cell progression	(Van den Berge et al., 2020; Wang et al., 2020)
Gene regulatory network	Celltalker, SCENIC, CellPhoneDB, CellChat	Gene regulatory network reconstruction, cell state analysis	(Vento-Tormo et al., 2018; Raredon et al., 2022)

As to the validation methods for key findings identified by single-cell sequencing, the specific cell subsets can be sorted and verified using flow cytometry (Lyu et al., 2025). Spatial transcriptomic techniques (e.g., 10× Visium) can be applied to validate the spatial distribution of gene expression revealed by single-cell sequencing, such as confirming the tissue localization of virus infection-related genes and examining whether the spatial patterns of cell–cell interactions agree with the predictions from single-cell sequencing (Millard et al., 2025). For the key virus infection-related genes screened by single-cell sequencing, qPCR and Western Blot can be used to verify their expression levels in infected and control groups, so as to confirm the reliability of the differential expression (McCarthy et al., 2017).

## 4 Applications of scRNA-seq in viral infection research

### 4.1 Identification of novel cell types

Viral infections can induce the formation of new cell subpopulations in the host, characterized by alterations in abundance, phenotype, and transcriptional profiles. These highly specific changes are critical for unraveling the mechanisms of viral pathogenesis and host responses. Traditional transcriptome sequencing methods tend to obscure the subtle changes in cell subpopulations. In contrast, scRNA-seq significantly enhances the possibility of identifying and characterizing unknown cell types, thereby enabling the capture of cell subpopulations with infection-specific alterations. Uyar et al. used scRNA-seq to identify a novel subpopulation of microglia termed “in transition” microglia/microglia-like cells in herpes simplex virus encephalitis (HSE). These newly discovered cells expressed genes such as Retnlg, Cxcr2 and I1159, which are typically associated with neutrophils functions, suggesting that these cells may exert neutrophil-like functions in immune and inflammatory responses. Moreover, these “in transition” microglia/microglia-like cells increased the production of IL-1β via activating the NLRP3 inflammasome pathway, thereby exacerbating neuroinflammation. The authors proposed that this unique microglial subset plays an important role in aggravating the inflammatory response and could serve as a potential therapeutic target for controlling neuroinflammation (Uyar et al., 2022). Another study employed scRNA-seq to investigate acute respiratory distress syndrome (ARDS) caused by SARS-CoV-2 infection and identified a new cell population labeled as “developing neutrophils”, which markedly elevated in number in ARDS patients. These cells expressed genes associated with neutrophil granule proteins, indicating that they may be functionally similar to neutrophils and potentially involved in the immune and inflammatory responses induced by SARS-CoV-2. The discovery of this novel cell population provides new insights into ARDS pathogenesis and facilitates the development of more effective treatment strategies (Wilk et al., 2020). Similarly, He et al. conducted an scRNA-seq analysis of memory B cells and plasma cells in convalescent COVID-19 patients, identifying a distinct subset of activated memory B cells. These cells are characterized by high expression of CD11c and CD95. Notably, this subset exhibited a significantly higher proportion of IgG (55.2%) and IgA (27.5%) antibody isotypes compared to classical memory B cells, which expressed only 12.0% IgG and 24.1% IgA. These findings suggest that the CD11c^high^CD95^high^ activated memory B cell subset may play a critical role in the humoral immune response against SARS-CoV-2 infection (He et al., 2021). The novel cell subsets and associated gene expression profiles identified by scRNA-seq have advanced our understanding of viral infections and offers valuable new insights and targets for viral infection control.

scRNA-seq overcomes the averaging effect of bulk transcriptomics and enables precise capture of specific novel cell subsets induced by viral infection. Through standardized quality control and batch correction strategies, it effectively avoids the interference of technical biases from multi-batch samples on biological signals. scRNA-seq provides a new perspective for in-depth dissection of viral pathogenesis and lays an experimental foundation for the formulation and optimization of personalized therapeutic regimens. However, this technique is limited by high sequencing costs and demanding bioinformatic analysis requirements. Furthermore, candidate mechanisms and key findings identified at the single-cell level must be further validated using cross-disciplinary techniques such as flow cytometry and spatial transcriptomics before they can be translated into reliable evidence for clinical applications.

### 4.2 Characterization of virus-induced specific gene expression profiles

Upon invading host cells, the components of the virus interact with various intracellular molecules, triggering extensive changes in gene expression. These alterations in genes not only affect the replication, assembly, and release of the virus but also impact the metabolism and cell cycle of the host cells. Hum et al. employed scRNA-seq to investigate microglial responses in the mouse brain following infection with Rift Valley fever virus (RVFV). They found that the expression levels of IFNβ1 and IFNα2 were significantly changed in microglia from wild-type mice, whereas in microglia from MAVS-deficient (MAVS^–/–^) mice, the expression of these two interferons was nearly undetectable. Gene Ontology (GO) analysis revealed enhanced antiviral response pathways in RVFV-infected microglia from wild-type mice. Whereas, microglia from MAVS^–/–^ mice exhibited a phenotype skewed toward pro-inflammatory responses, with increased activation of inflammatory signaling pathways. These findings highlight substantial gene expression changes in microglia upon RVFV infection and identify MAVS as a critical regulator of antiviral signaling in microglia, suggesting that MAVS is a potential therapeutic target for the prevention and treatment of RVFV infection (Hum et al., 2022). Feng et al. conducted a scRNA-seq analysis of hemocytes in Bombyx mori after infected with Bombyx mori nucleopolyhedrovirus (BmNPV). The analysis revealed that after BmNPV infection, most host genes were upregulated across various hemocyte populations, and most of these genes were associated with ribosomal pathways. While the expression of genes related to immune responses and RNA interference was generally suppressed, which may represent a viral strategy to enhance replication efficiency and evade host immune defenses (Feng et al., 2021).

An integrated analysis combining single-nucleus RNA sequencing, proteomics, and spatial transcriptomics of brainstem tissues from patients who died at different stages of COVID-19 was conducted to elucidate the mechanisms underlying neurological symptoms in the late stage of acute SARS-CoV-2 infection. They discovered that after infection, the immune-responsive glutamatergic neurons were primarily located in the dorsal motor nucleus of the vagus, while reactive microglia were widely distributed throughout the white matter region. Systemic inflammation caused by SARS-CoV-2 influenced different brain regions through two distinct mechanisms: one affecting specific areas of the brainstem and the other affecting the entire brainstem. In acute and late COVID-19, expression of proteins associated with neuronal and synaptic function, such as Akap7, Caprin1, and Atp2b, was significantly downregulated in the brainstem, potentially disrupting neurological structure and function. Furthermore, genes such as Isg15 and B2m were significantly upregulated in glutamatergic neurons, and Ifi44l and Ifi44 were strongly upregulated in microglia, macrophages, and endothelial cells, thereby inhibition of viral replication. Through the combined use of multiple omics technologies, this study provides profound insights into the spatial and cell-type-specific gene expression patterns in the brainstem during SARS-CoV-2 infection, offering valuable implications for developing therapeutic and preventive strategies targeting neurological complications of COVID-19 (Radke et al., 2024).

scRNA-seq overcomes the confounding effect of population-level gene expression averaging, delineates the specific transcriptomic profiles of distinct cell subtypes following viral infection, reveals cell-type-specific regulatory patterns of gene expression, and avoids the masking of critical signaling pathways imposed by bulk samples. The identified key infection-related genes contribute to the screening of specific therapeutic targets (e.g., key regulatory genes such as IFNβ1 and IFNα2), and support multi-omics integration to dissect complex pathological mechanisms and optimize therapeutic strategies. scRNA-seq relies on high-quality samples and multi-omics joint validation; its data interpretations require functional experimental support, and the capture sensitivity for low-abundance virus-induced genes remains to be further improved.

### 4.3 Identification of target cell types in viral infection

Accurate identification of viral target cells is critical for understanding viral replication and pathogenesis. Shah et al. found that Infectious Bursal Disease Virus (IBDV) primarily infects lymphoid tissues in the posterior nasal cavity. The scRNA-seq analysis further revealed that basal cells in the epithelium of bursa of Fabricius are recognized by viral particles by expressing KRT5 and TP63 proteins. The basal cells were identified as one of the main target cell types for IBDV. Moreover, IBDV infection resulted in a significant reduction in the number of IgM^+^ B cells, while the number of IgA^+^ B cells increased. This finding indicates a predominant mucosal immune response induced by IBDV, confirming that basal cells are among the main target cells of IBDV in the chicken bursa of Fabricius and uncovering the cellular antiviral mechanisms against IBDV infection (Shah et al., 2021). Martínez-Colón et al. detected SARS-CoV-2 RNA within adipocytes in COVID-19 autopsy samples, indicating direct infection of adipose tissue. They employed scRNA-seq to investigate the interaction between SARS-CoV-2 and human adipose tissue. The analysis identified adipocytes and a subset of inflammatory macrophages residing in adipose tissue as the primary target cell types of SARS-CoV-2 in adipose tissue. Intriguingly, they discovered that viral entry into adipocytes might occur through an ACE2-independent mechanism, suggesting alternative viral entry routes. These results imply that SARS-CoV-2 exacerbates the severity of COVID-19 by replicating within adipocytes and triggering inflammatory responses through the activation of resident macrophages in adipose tissue (Martínez-Colón et al., 2022).

scRNA-seq also enables the characterization of neural cell types targeted by viruses, offering distinct advantages in elucidating mechanisms of virus-induced neuropathology. In a study of Red-spotted Grouper Nervous Necrosis Virus (RGNNV) infection in the Grouper midbrain, scRNA-seq revealed that RGNNV infection induced an increase in macrophages and intensified cytokine production and inflammatory responses. Using scRNA-seq, 35 distinct cell subtypes were identified in the Grouper midbrain, including 28 neuronal subtypes and 7 non-neuronal types. Notably, RGNNV exhibited a preference for infecting specific neuronal subtypes, particularly the GLU1 and GLU3 glutamatergic neurons. It also identified the key genes and pathways involved in cytoplasmic vacuolization and autophagy, which are believed to be major contributors to RGNNV-induced neurons’ death (Wang Q. et al., 2021). Another study applied scRNA-seq to uncover the complex interactions and immune responses induced by Japanese Encephalitis Virus (JEV) in the central nervous system of infected mice. Notable changes were detected in the cellular composition of the brain post-infection. with The infiltration of the immune cells such as monocytes, macrophages, and T cells increased significantly, while the number of neurons and glial cells progressively decreased. JEV exhibited a strong tropism for specific neuronal subtypes, especially Baiap2^+^ neurons. Infection of these neurons led to extensive neural damage and triggered a robust inflammatory response (Yang et al., 2024).

scRNA-seq has overcome the critical limitation of traditional methods that fail to precisely identify viral target cells. It delineates the specific target cell subtypes of diverse viruses, reveals viral cell tropism, and characterizes the alterations in cellular composition and function following infection. This technology provides key insights into the mechanistic basis of viral pathogenesis, facilitates the identification of therapeutic targets directed at target cells or infection-related pathways, and supports the development of precise prevention and control strategies. Nonetheless, several limitations remain. Due to the inconsistency between transcript and protein levels, cell populations defined by transcriptomic marker genes are still subject to inaccuracy. It is therefore recommended to use multiple markers for cross-validation and to verify cell populations via experimental approaches such as IFA and flow cytometry.

### 4.4 Revealing host immune responses

Viral infections trigger complex innate and adaptive immune responses. scRNA-seq shows remarkable advantages in dissecting the intricate changes in immune cells and immune-related molecules. It has been widely applied in the analysis of immune mechanisms in COVID-19. Zhu et al. performed scRNA-seq on peripheral blood mononuclear cells (PBMCs) from patients infected with SARS-CoV-2 and influenza A virus, respectively. The results showed a significant increase in plasma cells in both infections, indicating that plasma cell expansion is a universal host defense mechanism against viral infections. In COVID-19 patients, the STAT1/IRF3 pathway was significantly activated in CD4^+^ T cells, whereas in influenza patients, the STAT3/NF-κB pathway was predominantly activated in CD4^+^ T cells, suggesting distinct immune response mechanisms induced by the two viruses. Moreover, COVID-19 patients exhibited elevate T cell apoptosisd, accompanied by increased expression of transcription factors related to pro-inflammatory cytokines, cytokine receptors, and interferon responses. In contrast, influenza patients showed enhanced activity of transcription factors related to pro-inflammation and host factors involved in virus-host interactions. This study highlights the unique challenges posed by SARS-CoV-2 and influenza A virus to the human immune system, underscoring the complexity and specificity of host-virus interactions (Zhu et al., 2020). Wen et al. conducted scRNA-seq analysis on PBMCs from convalescent COVID-19 patients and comprehensively revealed the changes in key immune cell subpopulations. During the early recovery phase, a significant reduction in T cell numbers was observed, along with an increase in classical CD14^++^ monocytes and high-level expression of inflammation-related genes. The study also identified novel sequence variations in specific regions of B cell receptors, such as IGHV3–23 and IGHV3-7, which is important for vaccine development. This study identifies potential molecular targets for vaccine and therapeutic antibody development from the perspective of immune response (Wen et al., 2020).

The immune mechanisms analysis of human cytomegalovirus (HCMV) in humanized mice was conducted by scRNA-seq HCMV infection significantly enhanced humoral immunity in mice, as evidenced by increased proportions of plasma cells, pre-B cells, and naive B cells; in contrast, cellular immunity was suppressed, as indicated by reduced proportions of memory CD8^+^ T cells and NKT cells. Plasma cells and pre-B cells showed upregulation of interferon-stimulated genes (e.g., IFI44, OAS1, ISG15, MX1), activating innate antiviral pathways. Memory CD8^+^ T cells downregulated ribosomal protein genes (RPL/RPS family) and molecular chaperones (e.g., HSP90), suggesting impaired protein synthesis function and antiviral effection. Interaction analysis using CellPhoneDB revealed that HCMV infection induced significant enhanced intercellular signaling among immune cells. By performing scRNA-seq on PBMCs from HCMV-infected humanized mice, this study uncovered key gene expression changes in different immune cells and providing insights into HCMV pathogenesis and potential therapeutic targets (Wang et al., 2024).

To elucidate the spatiotemporal dynamics of immune responses following viral infection, some studies have integrated multiple transcriptomic approaches. Kasmani et al. applied scRNA-seq, spatial transcriptomics, and bulk RNA sequencing to characterize immune responses to H1N1 influenza virus in mice of different ages. scRNA-seq revealed that, compared to 16-18-week-old young mice, 80-82-week-old aged mice exhibited upregulation of genes such as Tox, Eomes, and Casp3, associated with T cell activation, exhaustion, and apoptosis. The study identified three non-lymphoid endothelial cell populations: conventional endothelial cells, Vwf^+^ endothelial cells, and Car4^+^ endothelial cells. Car4^+^ endothelial cells in aged mice showed reduced vascular repair capacity and a pro-coagulant phenotype. Spatial transcriptomics showed that areas of severe lung fibrosis in aged mice exhibited higher levels of inflammation than those in young mice, indicating that inflammation may exacerbate pulmonary fibrosis. Neutrophils were more commonly located in the parenchymal regions of aged mouse lungs, potentially contributing to pathological lung injury. Differentially expressed genes in fibrotic vs. non-fibrotic regions included Wfdc17 (enriched in fibrotic areas) and Prss23 (enriched in non-fibrotic areas). Bulk RNA-seq revealed stronger inflammatory and fibrotic responses in the lungs of aged mice, with significant enrichment of pathways related to fatty acid metabolism and PPAR signaling. This study, through the integration of multi-omics technologies, comprehensively characterized the effects of influenza virus infection on mouse lungs, uncovering gene expression changes, spatial cell distribution, and activation states of various signaling pathways. These findings provide valuable insights into the disease progression of influenza in the elderly and offer broader implications for understanding respiratory viral infections (Kasmani et al., 2023). Liu et al. used scRNA-seq and single-cell VDJ sequencing (scVDJ-seq) to investigate the immune deficiency mechanisms in HIV-infected individuals with immune non-responders (INRs). The study analyzed 230,000 PBMCs from 33 individuals and identified 51 immune subpopulations. INR patients exhibited a significantly reduced proportion of CD4^+^ T cells and a markedly increased proportion of CD8^+^ T cells. scVDJ-seq further revealed that CD4^+^ T cells progressively differentiated into exhausted T cells, such as the CD4T_c08-TIGIT^+^ subset, accompanied by sustained activation of interferon signaling pathways. Using the scGeneANOVA algorithm, the study identified 33 INR-specific differentially expressed genes associated with T cell exhaustion. Viral load analysis via the VILDA tool demonstrated that INR patients harbored higher levels of HIV transcripts, which positively correlated with interferon signaling activation. This study, through the combined application of scRNA-seq and scVDJ-seq, for the first time elucidated that interferon-driven CD4^+^ T cell exhaustion is central to immune dysfunction in INR patients, providing a foundation for the development of precise immunotherapeutic strategies (Liu et al., 2025).

By investigating differences in viral replication across various host cell types, it is possible to clarify the patterns of viral replication and transmission *in vivo*, as well as the connection between these processes and the host’s immune response. Using scRNA-seq, Dai et al. analyzed the transcriptome 16 cell types of chicken lung tissue with highly pathogenic H5N1 avian influenza virus and low-pathogenic H9N2 avian influenza viruse, respectively. Compared to H9N2 infection, H5N1 infection induced stronger inflammatory and antiviral immune responses in pulmonary tissues. Following H5N1 infection, cells with high viral loads primarily included macrophage-like cells and M2-type macrophages. These cells produced large amounts of inflammatory cytokines (e.g., IFN-β, IL-1β, IL-6, and IL-8) after infection, which not only exacerbated immune-mediated lung damage but also facilitate further viral dissemination. The study further revealed a correlation between viral load and cellular responses: cells with high viral loads exhibited more intense pathological reactions and increased cell death, potentially explaining the greater disease severity associated with H5N1 infection (Dai et al., 2023). Zhu et al. employed scRNA-seq to investigate the intracellular distribution and replication dynamics of African swine fever virus (ASFV), finding that ASFV infection exerted significant effects on the number and function of cells of the porcine spleen. Viral load showed marked heterogeneity in monocytes and macrophages: 63.9% of monocytes and 16% of macrophages exhibited high viral loads. In the cell populations with high viral loads, interferon expression and antigen presentation capacity were significantly reduced. These findings suggest that ASFV may facilitate its replication by suppressing host antiviral responses. These studies highlight that scRNA-seq can reveal cell-type-specific heterogeneity in viral load and profound impact of viral load changes on cell function and immune response, providing a basis for exploring potential therapeutic targets (Zhu et al., 2024).

scRNA-seq overcomes the difficulty that the complex composition of immune cell populations makes targeted analysis challenging, enabling precise dissection of the subtype dynamics of innate and adaptive immune cells, the activation of specific signaling pathways, and the impact of viral load heterogeneity on immune function following viral infection. It also reveals the spatiotemporal characteristics of immune responses and the mechanisms of viral immune evasion. This technology provides critical evidence for elucidating the pathogenic mechanisms by which viruses mediate immune dysregulation, facilitates the identification of vaccine and therapeutic targets, and supports the development of precise immunotherapy strategies. However, several limitations remain: insufficient capture of low-abundance immune cell subsets; the requirement for multi-omics integration (including scVDJ-seq and spatial transcriptomics) and functional experiments to validate immune mechanisms; and high technical barriers in sample processing and bioinformatic analysis.

## 5 Challenges and future perspectives of scRNA-seq

### 5.1 Challenges and limitations

scRNA-seq imposes strict requirements on cell viability. During sample processing, cells may be damaged or killed due to tissue lysis, enzymatic digestion, or temperature fluctuations. These factors can compromise RNA quality and integrity, thereby affecting the accuracy and reliability of sequencing data (Macosko et al., 2015). Thus, a major challenge to scRNA-seq lies in isolating single cells from infected tissues while preserving RNA integrity. For certain tissues, such as the brain and spinal cord, the preparation and preservation of single-cell suspensions are particularly difficult. Most clinical samples are cryopreserved to maintain RNA stability, which limits the scRNA-seq studies on these valuable samples. Moreover, studies have shown that it tends to isolate easily dissociable cell types (e.g., immune cells), while cell types that are more fragile or difficult to dissociate, including endothelial and neuronal cells, are prone to loss or damage during the dissociation process. This incomplete capture of all diverse cell types in a tissue can lead to distorted cell proportions and reduce the accuracy of sequencing results in reflecting the true biological processes of viral infection (Garcia-Flores et al., 2023). The reduced cell viability and the greater difficulty in dissociating neural cells during neural tissue dissociation impose direct limitations on research related to neurotropic viruses. They lead to the selective loss of key viral target cells (such as neurons and endothelial cells), making it difficult to faithfully recapitulate the cellular compositional heterogeneity of neurotropic virus infections *in vivo*, and significantly interfere with the precise definition and quantitative analysis of viral cell tropism and tissue invasion range (Tang et al., 2025). It has been demonstrated that enzymatic dissociation at 37 °C can induce expression of cellular stress-response genes, leading to “artificial transcriptional stress responses” in transcriptional patterns and compromising the reliability of sequencing data (Adam et al., 2017; van den Brink et al., 2017). Cellular stress responses induced by tissue dissociation may mask the genuine early responses triggered by viral infection, which is particularly critical for studies focusing on the latent or early stages of infection (Ren et al., 2025).

Spatial information is critical for understanding how viruses spread within host tissues and trigger immune responses. However, scRNA-seq requires complete tissue digestion, which results in the loss of spatial information, preventing the identification of cell types and their gene expression profiles within the spatial context of the tissue (Denisenko et al., 2020). The loss of spatial information makes it difficult to accurately trace the transmission trajectory and diffusion route of viruses within host tissues, and precludes the systematic elucidation of the *in situ* regulatory interaction network between infected focus cells and immune cells, thereby significantly restricting the in-depth dissection of the molecular mechanisms underlying viral pathogenesis mediated by the local inflammatory microenvironment. Spatial stratification during sampling can provide broad spatial context for single-cell sequencing data; for instance, samples derived from distinct brain regions. Combined with spatial transcriptomics, such strategies can complement the spatial information of cell-specific or gene-specific expression (Li et al., 2026). Additionally, most current scRNA-seq technologies rely on poly(T) primers for reverse transcription, which limits sequencing to mRNA and lncRNA transcripts with poly(A) tails. For important transcripts lacking polyadenylation, such as certain lncRNAs and small RNAs, they can only be sequenced and analyzed through targeted amplification strategies (Kolodziejczyk et al., 2015). Therefore, for viruses whose transcripts lack poly(A) tails, only host responses can be analyzed, while viral transcription information is difficult to obtain, limiting the combined analysis of virus-host interactions.

### 5.2 Future prospects of scRNA-seq in viral infection research

With the advancement of artificial intelligence, more efficient algorithms and software tools are being developed for the analysis and mining of single-cell data. These tools will facilitate accurate predictions of viral transmission pathways and identification of potential antiviral drug targets. Moreover, the development of technologies capable of real-time monitoring of single-cell state dynamics would enable direct observation of the processes of viral entry, replication, assembly, and release, as well as the host cell’s immediate responses to different viral infection processes. During the viral attachment and entry phase, emerging real-time single-cell technologies are poised to enable direct visualization of the precise trafficking routes of viral particles-from receptor binding and intracellular transport to membrane fusion-thereby shedding light on how viruses will be unraveled to breach cellular barriers and initiate infection with unprecedented clarity (Reffsin et al., 2026). In the genome replication and gene expression phase, advances in real-time single-cell transcriptional dynamics are expected to capture the sequential activation of viral genes and host responses with heightened temporal resolution, paving the way for distinguishing abortive infection from productive replication more robustly and revealing previously uncharacterized kinetic bottlenecks that will shape our understanding of infection outcomes (Xiong et al., 2025). For the assembly and release phase, these evolving approaches hold great promise for monitoring the spatiotemporally coordinated processes of viral component assembly, budding, and release in real time, while uncovering the hidden heterogeneity in progeny yield and cell-to-cell transmission efficiency that population-level studies have thus far failed to capture (Eid et al., 2022). By preserving real-time kinetic characteristics and single-cell resolution, the next generation of dynamic single-cell methods will increasingly eliminate biases caused by population averaging, ultimately permitting comprehensive delineation of the sequential events, regulatory checkpoints, and heterogeneous mechanisms that govern the entire viral life cycle-filling critical gaps in our current knowledge and opening new avenues for targeted antiviral development (Ding et al., 2025). The integration of scRNA-seq with other technologies will also bring new breakthroughs for viral infection research. For example, Spatial transcriptomics can complement the lack of spatial information inherent in scRNA-seq. By combining spatial transcriptomics with scRNA-seq, individual cells within specific tissue regions could be localized and their gene expression profiles with spatial information could be analyzed, thereby deepening the understanding of the roles of individual cells during the infection (Vandereyken et al., 2023).

scRNA-seq also holds transformative potential for translational applications in antiviral immunity, including vaccine immunogenicity evaluation, immune memory profiling, and personalized immunotherapy. In vaccine development, scRNA-seq enables unbiased dissection of innate and adaptive immune cell dynamics, identifies rare antigen-specific cell subsets, and defines transcriptional signatures associated with high protective efficacy, thereby enabling rational optimization of antigen design and adjuvant formulation (Zelkoski et al., 2025). For immune memory analysis, longitudinal scRNA-seq coupled with TCR/BCR sequencing traces the generation, maintenance, and recall responses of long-lived memory T and B cells, clarifying the cellular basis of durable protection and guiding booster immunization strategies (Wen et al., 2025). In personalized immunotherapy, scRNA-seq delineates patient-specific immune landscapes, distinguishes responders from non-responders, and pinpoints dysfunctional or exhausted cell states, supporting the design of tailored immune interventions and combinatorial regimens to enhance antiviral and antitumor immunity. Together, these applications establish scRNA-seq as a cornerstone technology for advancing precision immunology and accelerating the clinical translation of next-generation antiviral vaccines and therapies.

## 6 Conclusion

**Figure 3:**
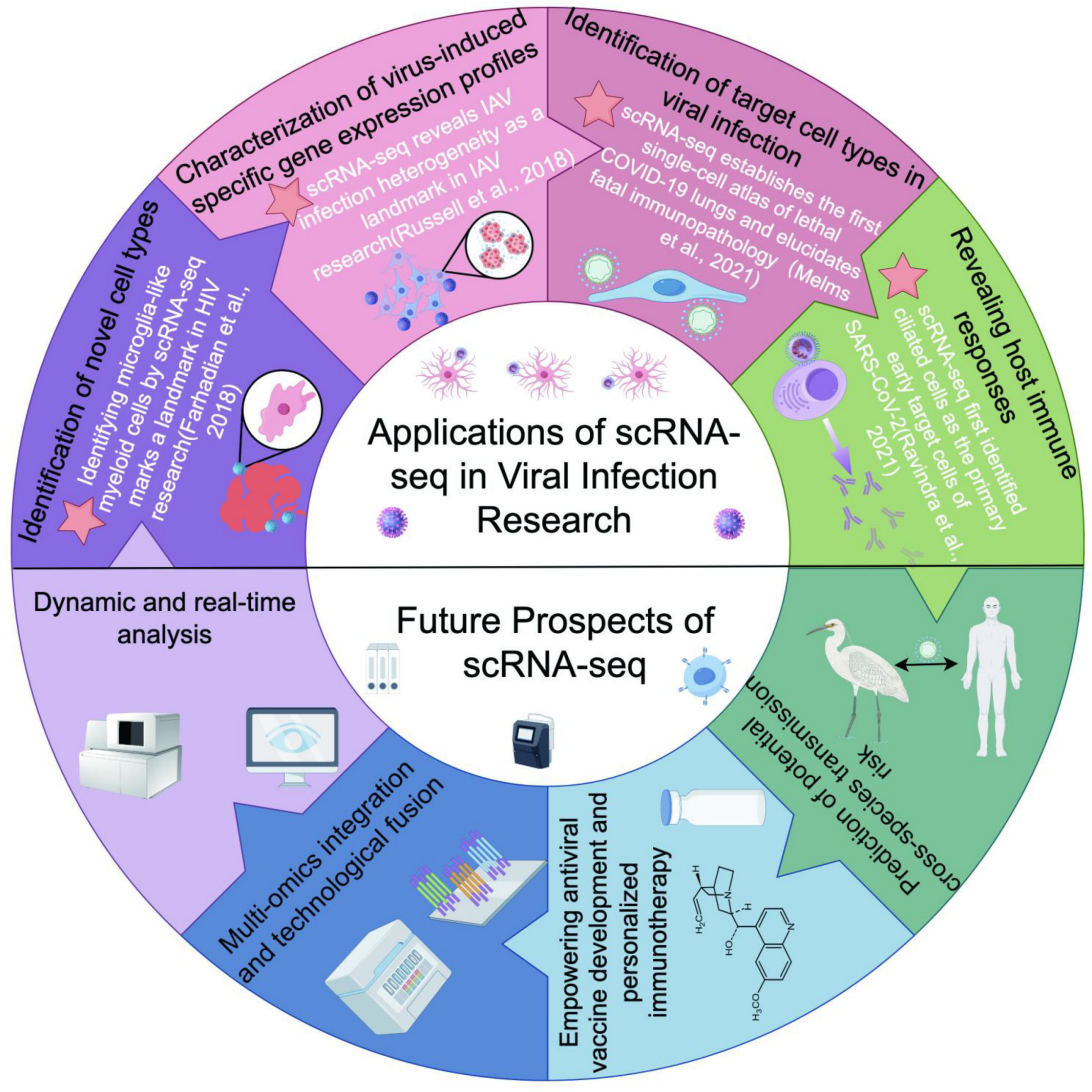
Key findings and future directions of scRNA-seq in viral infection research. Identifying microglia-like myeloid cells by scRNA-seq marks a landmark in HIV research (Farhadian et al., 2018). scRNA-seq reveals IAV infection heterogeneity as a landmark in IAV research (Russell et al., 2018). scRNA−seq establishes the first single-cell atlas of lethal COVID-19 lungs and elucidates fatal immunopathology (Melms et al., 2021). scRNA−seq first identified ciliated cells as the primary early target cells of SARS−CoV−2 (Ravindra et al., 2021). This schematic illustration was generated using Figdraw, and its reproduction in this work has been obtained with formal permission.

scRNA-seq has demonstrated tremendous potential in elucidating the complex virus-host interactions, significantly advancing our understanding of viral infections and overcoming several limitations of traditional transcriptome sequencing methods. scRNA-seq enables the identification of novel cell subtypes, the characterization of host immune responses, the analysis of changes in gene expression of specific cell subpopulations, and the determination of target cell types and *in vivo* distribution of viral infections, thereby providing crucial insights into viral pathogenesis (**Figure 3**). Moreover, scRNA-seq facilitates the development of broad-spectrum vaccines against diverse viral strains, the discovery of novel antiviral drug targets, and offers support for clinical diagnostics. Despite scRNA-seq still faces several challenges, such as high sequencing costs and the lack of spatial information, scRNA-seq has promising future iterations. It is expected to enable simultaneous detection of gene expression, protein changes, and epigenetic modifications, providing a more comprehensive understanding of viral infections.
